# Multitask Diffractive Deep Neural Networks Enabled by Joint Distance–Frequency Encoding

**DOI:** 10.1002/nap2.70342

**Published:** 2026-10-30

**Authors:** Zhengxuan Jiang, Zongmao Fei, Wen Jiang, Guowen Ding, Ke Chen, Yijun Feng

**Affiliations:** ^1^ Research Center of Applied Electromagnetics Nanjing University of Information Science and Technology Nanjing China; ^2^ School of Electronic Science and Engineering Nanjing University Nanjing China

**Keywords:** diffractive neural networks, distance–frequency encoding, metasurfaces, microwave computing, multi‐task learning

## Abstract

Diffractive deep neural networks (D^2^NNs) offer a promising route toward high‐speed wave‐based computing, but most existing architectures are restricted to a single fixed function, whereas multitask implementations often require additional hardware complexity. Here, a multitask D^2^NN architecture based on joint distance and frequency encoding is proposed and demonstrated. The interlayer distance is introduced as a physically reconfigurable task‐encoding dimension and is combined with a dual‐frequency phase‐independent metasurface to enable task multiplexing within a single passive hardware system. Scalar diffraction simulations on the corresponding testing datasets yielded accuracies of 99.7% for 9‐class orbital angular momentum (OAM) classification, 86.4% for 9‐class direction recognition, 98.8% for 4‐category programmable logic‐gate operations, and 95.4% for 4‐class MNIST digit recognition (Digits 0–3). Full‐wave electromagnetic simulations further support the physical feasibility of the designed metasurface implementation, whereas microwave measurements on selected representative inputs provide proof‐of‐concept evidence of task‐dependent energy routing and distance‐enabled task switching. This joint‐encoding strategy provides a compact and resource‐efficient route for multifunctional intelligent wave‐based computing systems.

## Graphical abstract

A joint distance–frequency encoding strategy enables a single passive diffractive neural network to switch among four computational tasks. By combining mechanically tunable layer spacing with a dual‐frequency phase‐independent metasurface, the same hardware performs orbital angular momentum classification, direction recognition, logic operations, and digit recognition, offering a compact route toward multifunctional wave‐based computing.

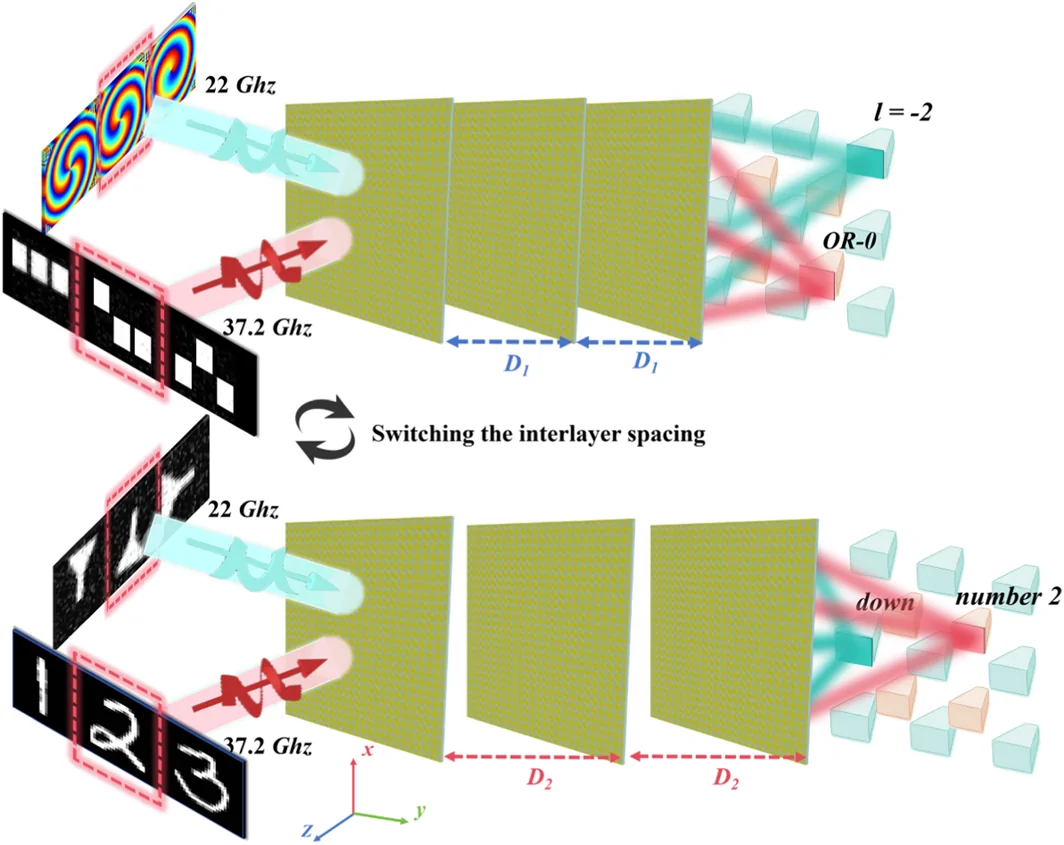

## 1 Introduction

As Moore's law gradually slows down, traditional electronic computing architectures are encountering fundamental bottlenecks in both computational power and energy efficiency, motivating the exploration of computing paradigms beyond the conventional von Neumann architecture. Photonic computing has emerged as a promising alternative, demonstrating tremendous potential for efficient artificial intelligence inference owing to its ultrahigh speed, low latency, and inherent parallelism [1]. Optical neural networks, in particular, leverage the wave nature of light to naturally perform large‐scale matrix operations, thereby significantly enhancing computational efficiency [2, 3]. Concurrently, advances in integrated photonic chips have enabled the realization of complex‐valued neural networks and reconfigurable diffractive processors, paving a viable pathway toward on‐chip optical computing [4]. Collectively, these developments have firmly established the strategic role of optical systems in next‐generation intelligent computing.

In recent years, diffractive deep neural networks (D^2^NNs) have garnered significant attention as an all‐optical computing architecture [5, 6]. This framework employs multiple trainable diffractive layers to establish end‐to‐end optical mappings, leveraging the wave nature of light to perform complex linear transformations directly at the physical level for specific intelligent tasks [7]. The fundamental principle of D^2^NNs lies in mapping neural network weights onto spatial modulation units within diffractive layers. After data‐driven training, the system can execute high‐speed inference on input optical fields without external power intervention, exhibiting unique advantages including passive operation, massive parallelism, and nanosecond‐scale response times [8]. Such networks have been successfully validated in applications such as image classification and object recognition [9], and further extended to optical logic operations [10] and image authentication systems [11], demonstrating their potential for multifunctional optical information processing. Moreover, researchers have explored deep‐tomographic holography techniques based on orbital angular momentum (OAM) mode multiplexing, which substantially enhances the system's multidimensional information processing capabilities [12]. With advances in integrated photonics, compact diffractive networks based on silicon metasurfaces have been progressively realized, accelerating the practical implementation of on‐chip optical neural networks [13]. In parallel, Xiong et al. demonstrated a reconfigurable diffractive processor based on cascaded metasurfaces, supporting classification, mathematical operations, and information encryption [14]. Related advances in phase‐only diffractive optics have also enabled the miniaturization of structured‐illumination imaging systems [15]. Multitask learning frameworks have also been introduced—for instance, functional multiplexing through multiwavelength inputs has preliminarily demonstrated the feasibility of supporting diverse functionalities on a single hardware platform [16]. Furthermore, Zang et al. demonstrated a detachable diffractive processor based on nonorthogonal polarization multiplexing, enabling multiple classification and holographic functions within a shared optical architecture [17]. Collectively, these advancements underscore that D^2^NNs not only possess formidable optical computing capabilities but also offer a viable pathway toward next‐generation low‐power, high‐speed intelligent systems. To further enhance hardware reusability and adaptability, researchers are now actively exploring reconfigurable diffractive network architectures.

As intelligent systems increasingly demand concurrent multitask processing capabilities, multifunctional integration has emerged as a pivotal strategy for enhancing the utilization efficiency of optical hardware [18]. Conventional diffractive deep neural networks are predominantly based on static designs, wherein functionalities—once physically encoded—become inflexible and ill‐suited for dynamically evolving task requirements, severely constraining their applicability in complex, real‐world scenarios [19]. Although strategies such as multiwavelength multiplexing have preliminarily enabled functional reuse on a single hardware platform, existing architectures still lack sufficient flexibility to support real‐time switching among diverse tasks [20]. To address this challenge, researchers have begun exploring reconfigurable optical neural network architectures that aim to achieve dynamic orchestration of multiple functions without increasing hardware complexity.

To overcome the functional limitations of static unit cells, researchers have explored diverse reconfigurable implementation strategies aimed at achieving multifunctionality through dynamic modulation of individual optical responses. Among these, active tuning mechanisms driven by external physical fields have emerged as the dominant approach. For instance, thermo‐optic phase reprogramming—achieved by modulating the refractive index of dielectric materials—has been successfully demonstrated in reconfigurable metalenses, showcasing promising capabilities in focal‐length tuning and wavefront shaping [21]. Similarly, electro‐optic control combined with phase‐change materials (e.g., Ge_2_Sb_2_Te_5_, GST) has enabled programmable metasurfaces capable of dynamically manipulating scattering patterns, polarization states, and focusing characteristics [22, 23]. Furthermore, the integration of electrical driving interfaces has led to highly efficient and stable phase control; notably, electrically driven phase‐change metasurfaces have also demonstrated pronounced optical modulation, with absolute reflectance contrasts reaching 80% [24]. In parallel, the fusion of multiplexing techniques with reconfigurable holographic imaging has established a new paradigm for multifunctional integration, supporting dynamic content switching and multimodal outputs [25].

Nevertheless, these approaches commonly rely on auxiliary components such as microheaters, bias circuits, or sophisticated feedback control systems, which inevitably increase architectural complexity and hinder high‐density integration and low‐power operation. This limitation becomes particularly pronounced in applications requiring frequent functional switching, where the latency and energy overhead introduced by peripheral driving circuitry are especially detrimental. In contrast, leveraging the intrinsic structural degrees of freedom of metasurfaces for efficient phase control offers a compelling alternative pathway toward system simplification.

Metasurface‐based phase control technologies provide a fundamental physical mechanism for realizing multifunctional optical systems. By meticulously engineering subwavelength unit cells, precise manipulation of electromagnetic wavefronts can be achieved on an ultrathin planar platform. Existing studies have demonstrated that, through the optimization of resonant modes, incorporation of multiorder resonant structures, or polarization‐division multiplexing, efficient wavefront shaping—including anomalous reflection, focusing, multibeam radiation, and vortex‐beam generation—can be realized using compact metasurface platforms [26, 27, 28, 29]. For example, coding metasurfaces combined with phase‐gradient designs have successfully enabled independent beam steering and OAM state generation at distinct frequency channels [30, 31], whereas dual‐band metalenses based on graphene or multilayer stacked architectures have exhibited robust focusing performance [32, 33].

Moreover, low cross talk and wide phase coverage (approaching 2π) are critical metrics for ensuring high system efficiency and output fidelity; these have been significantly enhanced in recent years through joint amplitude‐and‐phase modulation strategies [34]. Deep learning–assisted inverse design methodologies have further accelerated the realization of complex multifrequency responses, enabling independent generation and control of four‐channel vortex beams [35].

Beyond these advances in multifrequency phase engineering, recent metasurface and diffractive‐neural‐network platforms have further expanded the dimensionality of wave‐based information processing. For example, Zeng et al. combined the radial and azimuthal indices of Laguerre–Gaussian modes, the rotational states of cascaded diffractive layers, and input polarization to realize a 96‐channel multiplexed holographic system [36]. Chen et al. employed spin‐decoupled multilayer phase modulation to realize 12‐channel OAM–wavelength–polarization multiplexing and demultiplexing using a five‐layer diffractive metasurface [37]. More recently, wavelength‐dependent rotatable diffractive deep neural networks enabled selectable cross‐transformations among OAM–wavelength channels for dynamic optical interconnection [38]. Tan et al. further demonstrated a multidimensional reconfigurable diffractive network supporting 120 high‐fidelity optical multiplexing channels using only four metasurface layers and a compact meta‐atom set [39]. These studies demonstrate impressive channel capacity and flexible wave transformations, primarily for holographic imaging and mode‐multiplexed optical communication. Their architectural objective is mainly to enlarge the number of multiplexed channels or to reconfigure transformations within a related functional framework. In contrast, this work targets heterogeneous task‐level reconfiguration. Its architectural distinction lies not in maximizing channel capacity but in using frequency and axial interlayer distance as complementary task‐encoding variables, such that the same passive diffractive hardware can support fundamentally different computational functions.

From this perspective, system geometry, particularly the interlayer spacing, remains insufficiently explored as an explicit functional encoding dimension in diffractive deep neural networks. Mechanical tuning offers an alternative low‐power and zero‐static‐power reconfiguration pathway for optical systems. Adjusting interlayer spacing via microelectromechanical systems (MEMS) or similar actuation mechanisms has been shown to effectively alter optical field propagation characteristics and interference responses [40]. As reviewed by Zang et al., mechanically driven reconfigurable metasurfaces exhibit excellent flexibility and stability, opening new avenues for dynamic photonic devices [41]. Crucially, variations in interlayer distance significantly modulate the spatial interference patterns of diffracted fields, thereby naturally introducing a new degree of freedom for task encoding. However, this geometric parameter has rarely been jointly exploited with frequency within a unified task‐mapping framework, limiting its potential for heterogeneous multifunctional computing.

Addressing the aforementioned challenges and technological limitations, this work presents a novel distance‐encoded diffractive deep neural network architecture (DD^2^NN) that transcends the functional singularity of conventional diffractive networks by synergistically leveraging interlayer spacing and dual‐frequency control as complementary task‐encoding dimensions. The core innovation lies in establishing a two‐dimensional frequency–distance task‐mapping mechanism, wherein mechanically tunable interlayer distance serves as an independent and highly efficient encoding variable, jointly enabling decoupled multitask control with the spectral degree of freedom.

Specifically, we design a metasurface capable of independent phase modulation at two distinct frequencies, thereby establishing two frequency‐selective electromagnetic computing channels. Furthermore, we propose a shared‐phase joint optimization algorithm that effectively reconciles phase constraints across diverse tasks. This enables the coexistence of four fundamentally different functionalities—OAM mode classification, motion direction recognition, programmable logic‐gate operations, and MNIST handwritten digit recognition—on a single hardware platform. This approach offers a potentially useful framework for developing compact, multifunctional wave‐based computing systems.

## 2 Design and Principle

Our joint‐encoding architecture, shown schematically in Figure 1, is built upon a foundation of shared‐phase diffractive layers but introduces two dimensions for task multiplexing: frequency and physical distance. The system is designed to operate with a dual‐frequency source (*f*_1_ and *f*_2_). These frequencies pass through the same physical metasurface layers, which are engineered to provide independent phase responses.

**FIGURE 1:**
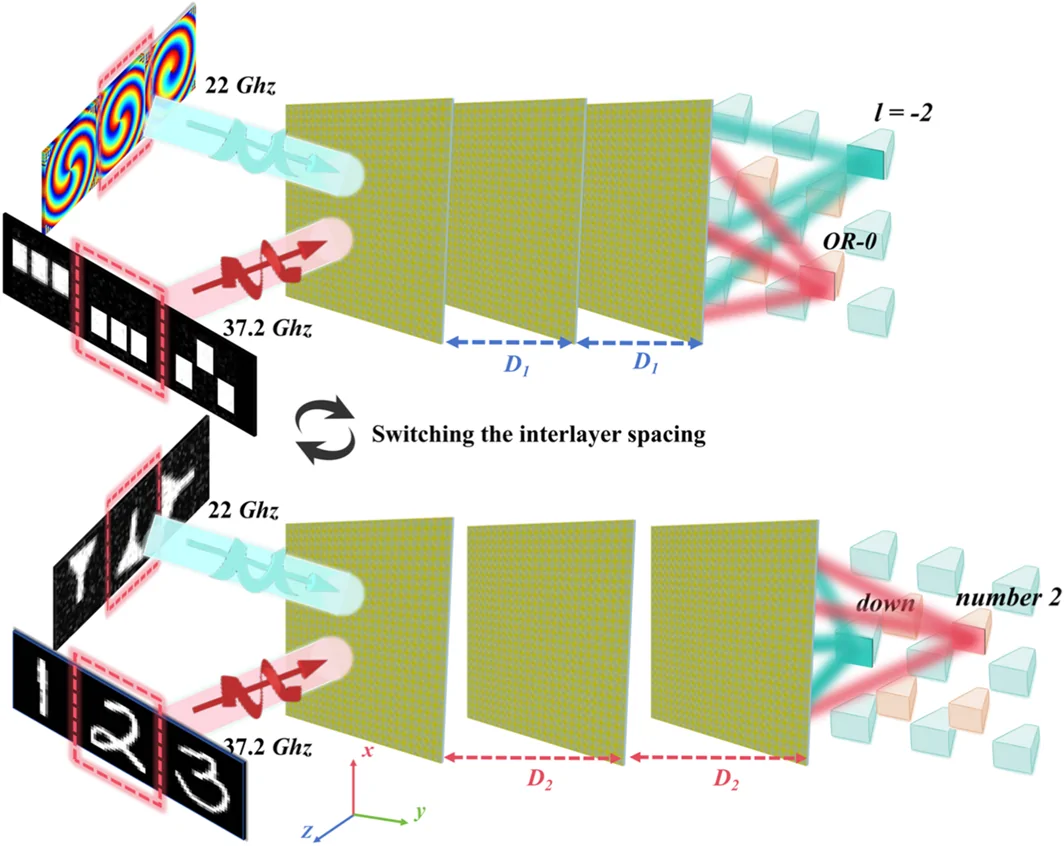
Schematic diagram of the distance‐encoded diffractive deep neural network (DD^2^NN).

Crucially, we introduce the interlayer spacing as a physically reconfigurable parameter, allowing the system to switch between two distinct distance configurations (*d*_1_ and *d*_2_). This creates a 2 × 2 task matrix where a single set of passive diffractive layers can execute four independent tasks. The four frequency–distance combinations and their corresponding computational tasks are summarized in Table 1. These tasks are evaluated in Section 4 through scalar diffraction and full‐wave electromagnetic simulations, supplemented by representative proof‐of‐concept microwave measurements. This approach multiplexes tasks without requiring any change to the static, passive phase elements themselves.

**TABLE 1:** Frequency–distance task mapping of the proposed DD^2^NN.

Operating frequency	*d*_1_ = 60 mm	*d*_2_ = 80 mm
*f*_1_ = 22 GHz	OAM classification (9 classes)	Direction recognition (9 classes)
*f*_2_ = 37.2 GHz	Logic‐gate operations (4 categories)	MNIST recognition (4 classes)

The proposed DD^2^NN employs two operating frequencies (*f*_1_ = 22 GHz and *f*_2_ = 37.2 GHz) and two uniform interlayer‐distance configurations (*d*_1_ = 60 mm and *d*_2_ = 80 mm), forming four distinct frequency–distance task states. As summarized in Table 1, the (*f*_1_, *d*_1_) and (*f*_1_, *d*_2_) configurations are assigned to 9‐class OAM classification and 9‐class direction recognition, respectively, whereas the (*f*_2_, *d*_1_) and (*f*_2_, *d*_2_) configurations correspond to 4‐category logic‐gate operations and 4‐class MNIST digit recognition, respectively. The distance values represent the uniform spacing between adjacent diffractive planes rather than the total propagation length of the network.

The physical basis for our distance encoding lies in the fundamental principles of wave propagation. The complex field *u* ^(*l*+1)^ at layer *l* + 1 is related to the field at layer *l* through scalar diffraction, which is modeled using a simplified Rayleigh–Sommerfeld‐based propagation kernel [42]. This propagation is described by a transfer matrix ***W***_*l*,*l*+1_, where each element *w*_*l*,*l*+1_ (*i*, *j*), connecting a neuron *j* to a neuron *i*, is a strong function of the propagation distance *d*_*l*,*l*+1_ (the distance between layers *l* and *l* + 1) and the wavenumber *k* = 2π *f*/*c*.

The propagation matrix element can be expressed as follows [43]:

(1) $$w_{l,l+1}\left(i,j\right)=\frac{\exp\left(-jkr_{ij}\right)}{r_{i,j}^{2}}$$

where $r_{i,j}=\sqrt{{\left(x_{i}-x_{j}\right)}^{2}+{\left(y_{i}-y_{j}\right)}^{2}+d_{l,l+1}^{2}\;}$

Any change in the layer distance *d*_*l*,*l*+1_ directly and nonlinearly alters this complex‐valued transfer function. In the paraxial approximation, this effect is often captured by the Fresnel diffraction kernel [44, 45]:

(2) $$\exp\left(-jkr_{ij}\right)\approx \exp\left(-jkd_{l,l+1}\right)\exp\left(-jk\frac{{\left(x_{i}-x_{j}\right)}^{2}+{\left(y_{i}-y_{j}\right)}^{2}}{2d_{l,l+1}}\right)$$

This clearly shows that the distance *d*_*l,l+*1_ modulates both the overall phase exp(−*jkd*_*l*,*l+*1_) and the spatial‐frequency‐dependent Fresnel diffraction term.

In a D^2^NN, this free‐space propagation ***W*** is combined with the static modulation from the diffractive layers. We can physically represent the phase modulation of each layer *l* as a complex‐valued transmission matrix ***M*** ^(*l*)^. The total transformation implemented by the network is therefore a combination of both this static set of phase matrices {***M*** ^(*l*)^} and the dynamic, distance‐dependent propagation matrices {***W*** (*d*_*j*_)}. Consequently, two configurations of the same network built from the exact same set of physical phase layers and thus identical transmission matrices {***M*** ^(*l*)^} but configured with different layer spacings (*d*_1_ vs. *d*_2_) will implement two fundamentally different complex‐valued transformations, *T*_1_(*u*) ≈ ***M*** ^(*l+*1)^ ⊙ (***W***_(*l*,*l+*1)_ (*d*_1_)*u*) and *T*_2_(*u*) ≈ ***M*** ^(*l+*1)^ ⊙ (***W***_(*l,l+*1)_ (*d*_2_)*u*), where *u* denotes the incident field, ***M*** ^(*l+*1)^ represents the shared phase‐modulation matrix of layer *l* + 1, and ***W***_(*l,l+*1)_ (*d*_1_) and ***W***_(*l,l+*1)_ (*d*_2_) are the propagation operators corresponding to the two distance configurations. Here, *T*_1_ and *T*_2_ denote the resulting layerwise transformations.

Our multitask learning algorithm, summarized in Figure 2, trains a single set of shared phase‐only modulation layers {***M*** ^(*l*)^} to perform *N* different tasks across *K* frequencies and *I* distance configurations. For clarity, the following mathematical formulation describes the joint optimization of distance‐encoded tasks at a fixed operating frequency.

**FIGURE 2:**
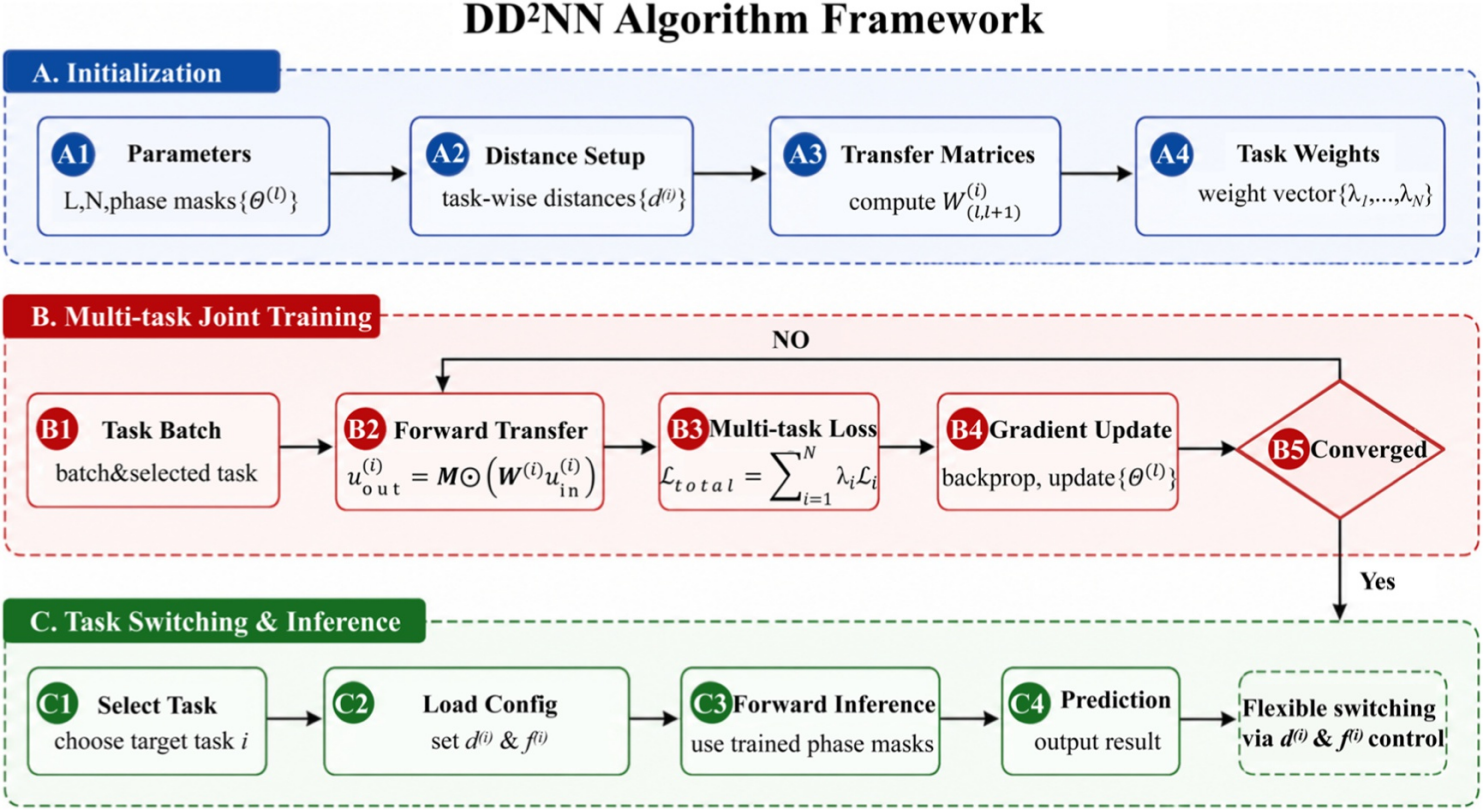
DD^2^NN algorithm framework.

The key innovation is that although the phase matrices ***M*** ^(*l*)^ are static and shared, the propagation matrix ***W***_(*l,l+*1)_ is dynamic and task‐dependent. For a specific task *i* (e.g., Task A, *f*_1_/*d*_1_), the network uses a precalculated propagation matrix $W_{\left(l,l+1\right)}^{A}$ corresponding to its specific frequency *f*_1_ and distance configuration *d*_1_.

To train the shared phase parameters, we employ a weighted multitask loss function defined as follows:

(3) $$L_{\text{total}}=\sum_{i=1}^{N}\lambda_{i}L_{i}$$

where $L_{i}$ represents the loss function of task *i* and $\lambda_{i}$ denotes its corresponding weighting coefficient. For classification tasks, the loss function is based on the cross‐entropy between the predicted output distribution and the target labels.

For a phase‐only diffractive layer (*l*), the complex transmission matrix is expressed as $M^{\left(l\right)}=\exp\left(j\Theta^{\left(l\right)}\right)$, where $\Theta^{\left(l\right)}$ is the real‐valued trainable phase matrix and the exponential is evaluated elementwise. The shared phase parameters are updated by minimizing the total multitask loss using the Adam optimizer:

(4) $$\Theta_{t+1}^{\left(l\right)}=\Theta_{t}^{\left(l\right)}-\eta_{t}\;\text{Adam}\left(\nabla_{\Theta_{t}^{\left(l\right)}}L_{\text{total}}\right)$$

where *t* is the optimization iteration, $\eta_{t}$ is the learning rate, and Adam(·) denotes the adaptive update direction obtained from the gradient and its accumulated first‐ and second‐moment estimates. Optimizing the real‐valued phase matrix rather than directly updating the complex transmission coefficients preserves the phase‐only modulation constraint.

During joint training, the phase parameters are shared among the tasks within each frequency‐specific training group, whereas the propagation operators depend on the selected operating frequency and interlayer‐distance configuration. Consequently, the optimization identifies a common set of phase profiles capable of supporting different task‐dependent input–output transformations without modifying the physical phase layers during inference.

To enhance the robustness of the trained network against practical assembly errors, alignment‐aware perturbation augmentation was incorporated into the joint training process. During training, the three internal diffractive layers were independently subjected to randomly sampled longitudinal and lateral displacements. The maximum longitudinal perturbation was set to ± 2.0 mm, whereas the maximum displacement along each transverse direction was set to ± 1.0 mm. The alignment perturbations were applied with a probability of 0.8, and their maximum ranges were gradually introduced during the first 100 training iterations. For each perturbed forward pass, the propagation process was evaluated using the displaced layer geometry, whereas the shared phase parameters were jointly optimized for the two tasks in the corresponding frequency channel. It should be emphasized that these perturbation ranges are training‐augmentation parameters rather than the measured positioning tolerances of the final network.

Once trained, task switching does not require retraining or reprogramming the passive diffractive layers. During inference, the desired task is selected by setting the excitation frequency to the corresponding operating frequency and mechanically adjusting the uniform interlayer spacing to the predefined distance configuration. The resulting electromagnetic field propagation is determined by the task‐dependent propagation operators ***W***_(*l,l+*1)_ and the fixed phase profiles encoded in the physical metasurface layers. Therefore, different computational functions can be realized using the same passive hardware through joint frequency–distance selection, without modifying the fabricated phase patterns.

## 3 Design of the Dual‐Frequency Phase‐Independent Metasurface

To physically implement the proposed joint‐encoding architecture, a key hardware requirement is a diffractive surface capable of providing independent phase modulation at two distinct frequencies (*f*_1_ and *f*_2_) within a single passive structure. The proposed unit cell, illustrated in Figure 3a, utilizes the geometric‐phase (Pancharatnam–Berry phase) principle. It features a three‐layer metal–dielectric–metal “sandwich” architecture with a periodicity of *p* = 4 mm. The metallic patterns are modeled as gold with a conductivity of 4.56 × 10^7^ S/m and a thickness of 0.035 mm. The fabricated prototype follows the same metal–dielectric–metal geometry using metallization compatible with printed circuit board fabrication.

**FIGURE 3:**
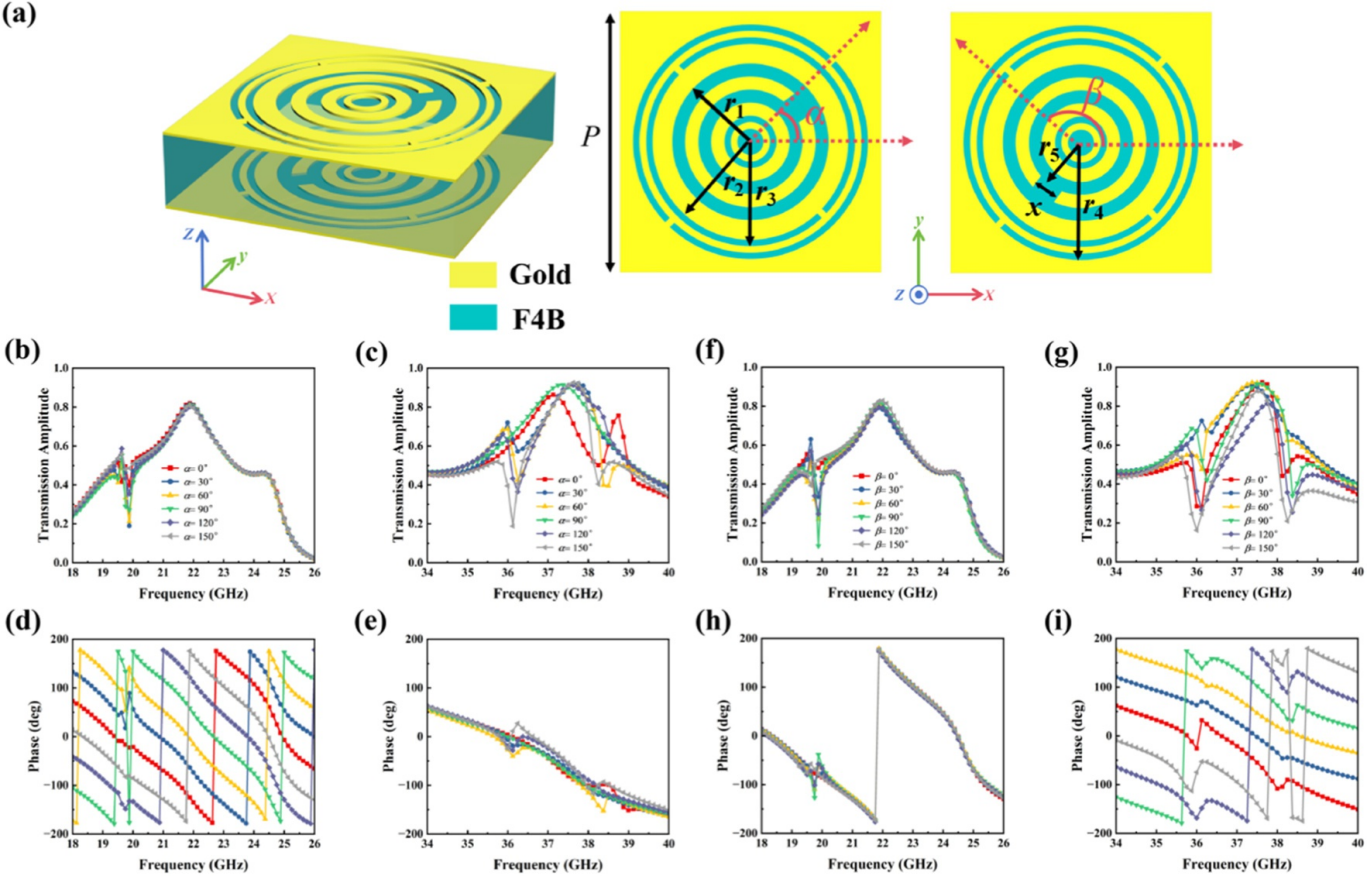
Dual‐frequency metasurface unit cell and transmission characteristics. (a) Unit‐cell structure with outer DSRR and inner SRR. (b–e) Transmission amplitudes (b, c) and phases (d, e) under outer rotation *α*. (f–i) Corresponding amplitudes (f, g) and phases (h, i) under inner rotation *β*.

The top functional layer consists of a composite structure comprising an outer double split‐ring resonator (DSRR) nested with an inner split‐ring resonator (SRR). The outer DSRR is formed by etching two sets of concentric split‐ring slots with different diameters into the metal layer, characterized by radii *r*_3_ through *r*_2_. The inner SRR is defined by radius *r*_5_ with a gap width of *x*. The bottom metallic layer mirrors the top geometry but with the inner SRR orientation rotated by 180° to enhance cross‐polarization conversion efficiency. The middle layer serves as a spacer, composed of a 1.5‐mm‐thick F4B dielectric substrate with a dielectric constant of 2.65 and a loss tangent of 0.001.

Crucially, this design provides two independent rotational degrees of freedom: the rotation angle *α* of the outer DSRR and the rotation angle *β* of the inner SRR, which control the phase responses at 22 and 37.2 GHz, respectively.

As shown in Figure 3b–e, varying the outer DSRR rotation angle *α* induces a full 2π phase shift in the low‐frequency band while leaving the high‐frequency response virtually unaffected. Conversely, Figure 3f–i demonstrates that rotating the inner SRR by angle *β* modulates the phase in the high‐frequency band across the full 2π range, with minimal impact on the low‐frequency band. This approximate phase decoupling confirms that the phase profiles at *f*_1_ and *f*_2_ are independently controlled by *α* and *β*, respectively, allowing us to encode arbitrary, task‐specific phase maps for both frequencies onto a single physical surface. A detailed analysis of the rotation‐angle‐dependent phase linearity, including the phase‐unwrapping and linear‐fitting procedures, is provided in Supporting Information S1: Note S2.

Based on this decoupling principle, the physical realization of the proposed DD^2^NN layers follows the mapping strategy illustrated in Figure 4. First, four distinct tasks are grouped into two frequency channels: Task 1 (MNIST digit recognition) and Task 2 (logic‐gate operations) share the high frequency *f*_2_ (37.2 GHz), whereas Task 3 (OAM classification) and Task 4 (direction recognition) share the low frequency *f*_1_ (22 GHz). The multitask learning algorithm then generates two independent phase distributions, $\Phi_{1}\left(x,y\right)$ and $\Phi_{2}\left(x,y\right)$, optimized for the task pairs at each frequency.

**FIGURE 4:**
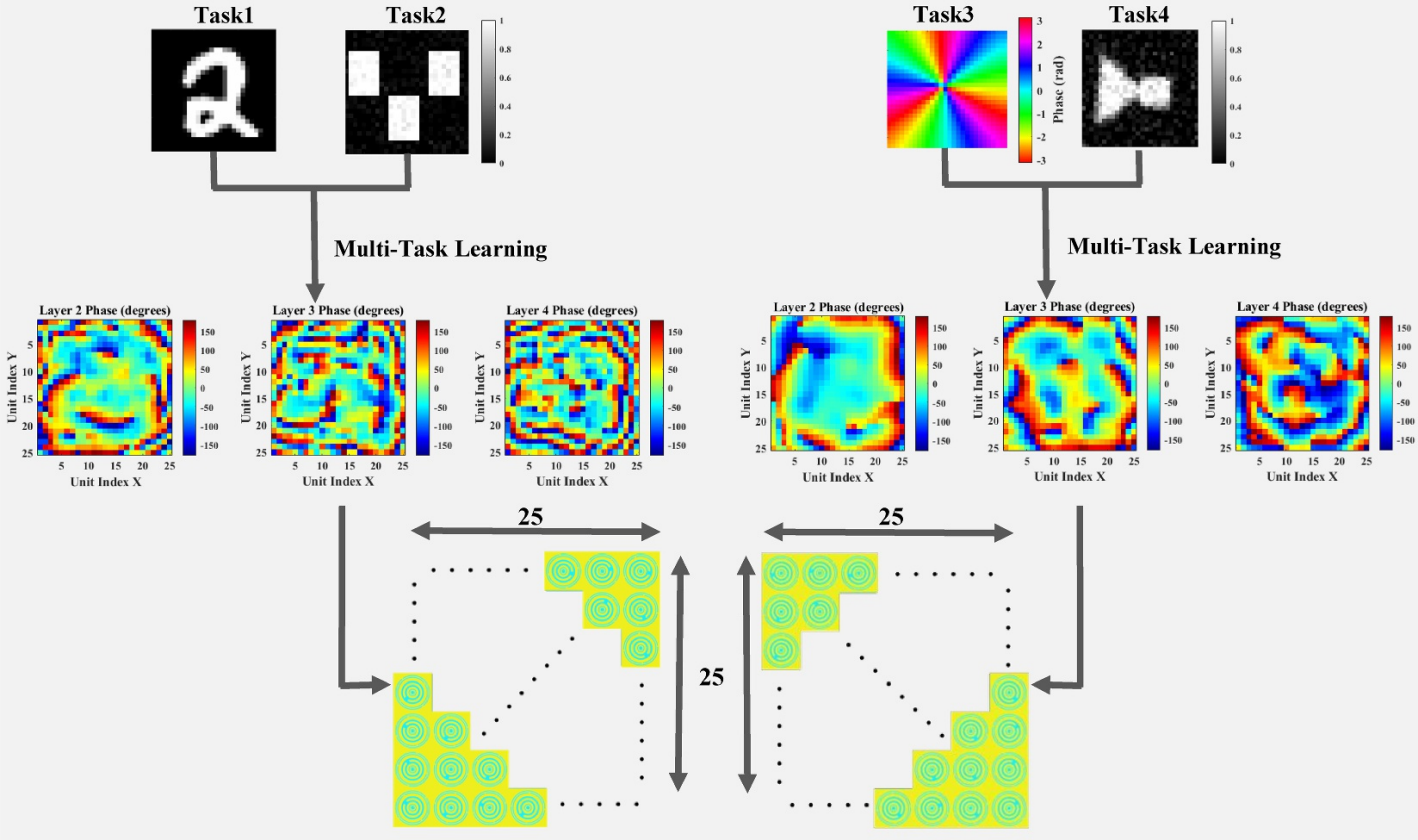
Multitask learning and shared‐phase metasurface construction. Two frequency‐specific dual‐task groups are independently optimized to generate phase profiles at 22 and 37.2 GHz, which are subsequently integrated into the same physical metasurface layers.

According to the Pancharatnam–Berry geometric‐phase principle, the circular‐polarization‐converted transmission component acquires an ideal phase shift of $\Delta \Phi_{PB}=\pm 2\theta$ when an anisotropic structure is rotated by an angle *θ*. Consequently, under the adopted polarization and phase‐reference conventions, the required physical rotation angle is one half of the target phase modulation. A detailed theoretical derivation is provided in Supporting Information S1: Note S3. Accordingly, the optimized phase distributions are mapped to the rotation angles of the outer DSRR and inner SRR as follows:

(5) $$\alpha \left(x,y\right)=\frac{\Phi_{1}\left(x,y\right)}{2},\beta \left(x,y\right)=\frac{\Phi_{2}\left(x,y\right)}{2}$$

As shown in the lower panel of Figure 4, the dual‐frequency phase profiles are integrated into a single 25 × 25 metasurface array, where each unit cell encodes independent phase values for both frequencies through the two rotational degrees of freedom (*α* and *β*). By assembling these unit cells, we construct a dual‐frequency phase‐independent metasurface that serves as the shared physical hardware for the entire multitask system.

## 4 Results and Analysis

To evaluate the proposed DD^2^NN architecture, we adopted a three‐level verification strategy, including scalar diffraction modeling, full‐wave electromagnetic simulation, and representative microwave measurements.

We first conducted numerical simulations in MATLAB using a 5‐layer DD^2^NN structure (including input layer, output layer, and three intermediate phase‐modulation layers). Two simulation groups were designed to evaluate the multitask switching capability.

The first group focuses on logic‐gate operations and MNIST classification at *f*_2_ = 37.2 GHz. The network configuration was [28 × 28, 25 × 25, 25 × 25, 25 × 25, 2 × 2] units with physical widths of [3.3, 4, 4, 4, 50] mm. The interlayer spacing was set to [60, 60, 60, 60] mm for logic‐gate operations and [80, 80, 80, 80] mm for MNIST classification. The logic‐gate dataset contains four operation modes (AND‐1, AND‐0, OR‐1, and OR‐0), whereas the MNIST dataset includes Digits 0–3, each with 5000 samples per class.

The second group addresses OAM and directional classification at *f*_1_ = 22 GHz. The network configuration was [28 × 28, 25 × 25, 25 × 25, 25 × 25, 3 × 3] units with physical widths of [3.3, 4, 4, 4, 33] mm. The interlayer spacing was [60, 60, 60, 60] mm for OAM classification and [80, 80, 80, 80] mm for direction recognition. The OAM dataset contains 9 modes (*l* = −4 to +4), and the directional dataset includes 9 categories, each with 5000 samples. All datasets were split at an 8:2 ratio for training and testing.

Both groups employed the Adam optimizer with an initial learning rate of 0.15, decayed by 50% every 20 epochs. The output category was determined by the detector region with the maximum normalized energy. The classification accuracy was calculated over the testing set. Task weights were set to [0.3, 0.7] to prioritize the more challenging second task. Detailed descriptions of dataset construction, sample variability, and train–test separation for the OAM‐classification, direction‐recognition, and logic‐gate tasks are provided in Supporting Information S1: Note S1.

The numerical simulation results validate the effectiveness of the proposed DD^2^NN architecture across all four tasks. At *f*_2_ = 37.2 GHz, the network achieves high‐confidence classification for MNIST Digits 0–3, with distinct energy concentration at the correct output positions and overall accuracy exceeding 95%, as shown in Figure 5a. The logic‐gate operations demonstrate similar performance, correctly implementing all four modes (AND‐1, AND‐0, OR‐1, and OR‐0) with clear discrimination between different logic states (Figure 5b).

**FIGURE 5:**
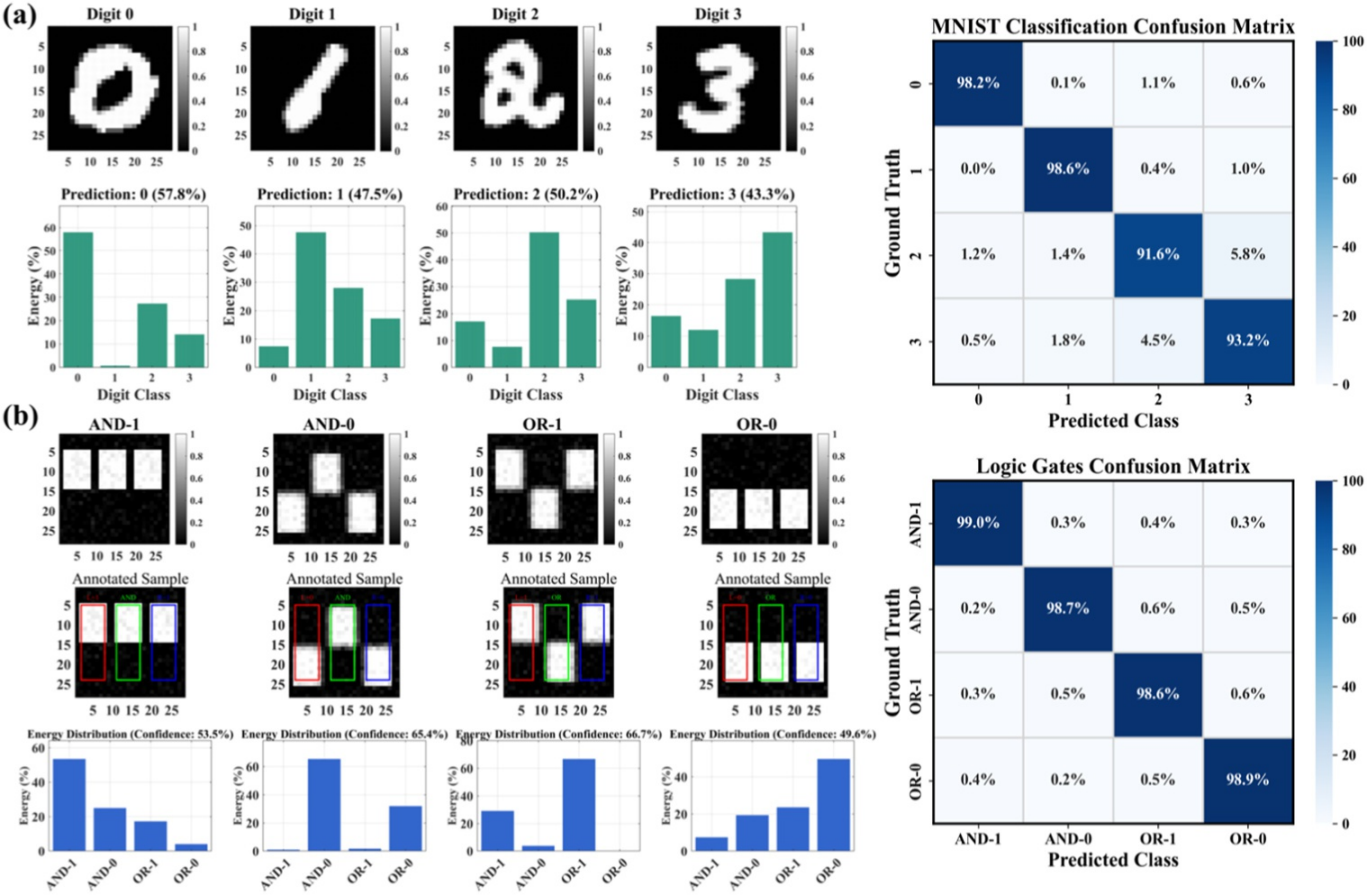
Numerical simulation results at 37.2 GHz. (a) MNIST digit recognition (0–3): input samples, output energy distributions, and confusion matrix. (b) Logic‐gate operations (AND‐1, AND‐0, OR‐1, and OR‐0): input patterns, output energy distributions, and confusion matrix.

At *f*_1_ = 22 GHz, the OAM classification task achieves a remarkable accuracy of 99.7% in distinguishing 9 orbital angular momentum modes (*l* = −4 to +4), as evidenced by the near‐diagonal confusion matrix in Figure 6a. The direction recognition task successfully identifies 9 movement categories with 86.4% accuracy (Figure 6b), where the slightly lower performance is attributed to inherent similarities between adjacent directional patterns.

**FIGURE 6:**
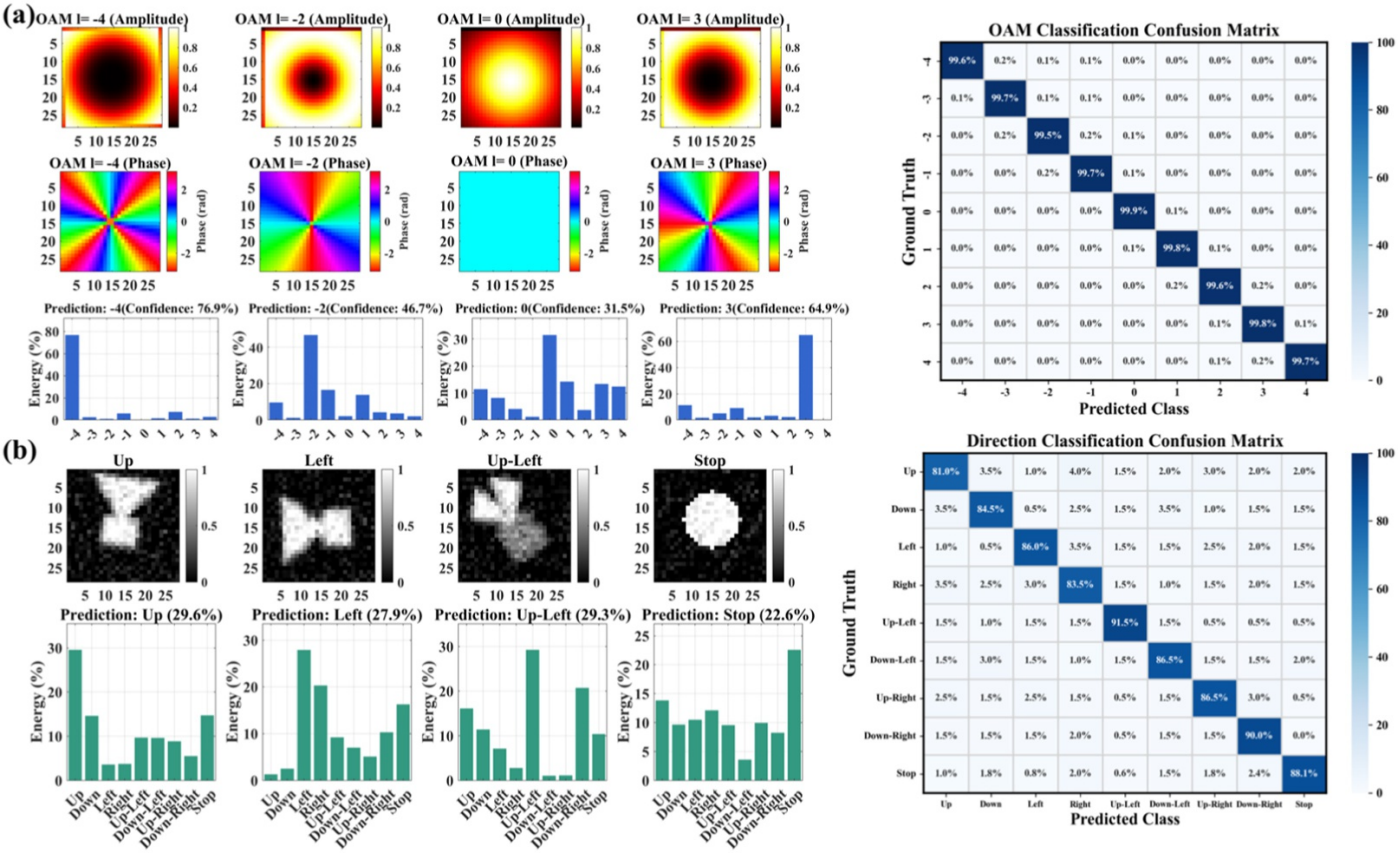
Numerical simulation results at 22 GHz. (a) OAM classification (*l* = −4 to +4): sample field profiles, output energy distributions, and confusion matrix. (b) Direction recognition (9 categories): input patterns, outputs, energy distributions, and confusion matrix.

Crucially, the task‐switching capability enabled by interlayer spacing reconfiguration is demonstrated in Figure 7. When identical inputs are processed at the same frequency but with different layer spacings, the network produces fundamentally different output distributions corresponding to distinct tasks. As shown in Figure 7a, switching from *d*_1_ to *d*_2_ at *f*_2_ = 37.2 GHz transforms the network function from logic‐gate operations to MNIST classification. Similarly, Figure 7b confirms that the same mechanism operates at *f*_1_ = 22 GHz, validating interlayer distance as an effective encoding dimension for multitask switching.

**FIGURE 7:**
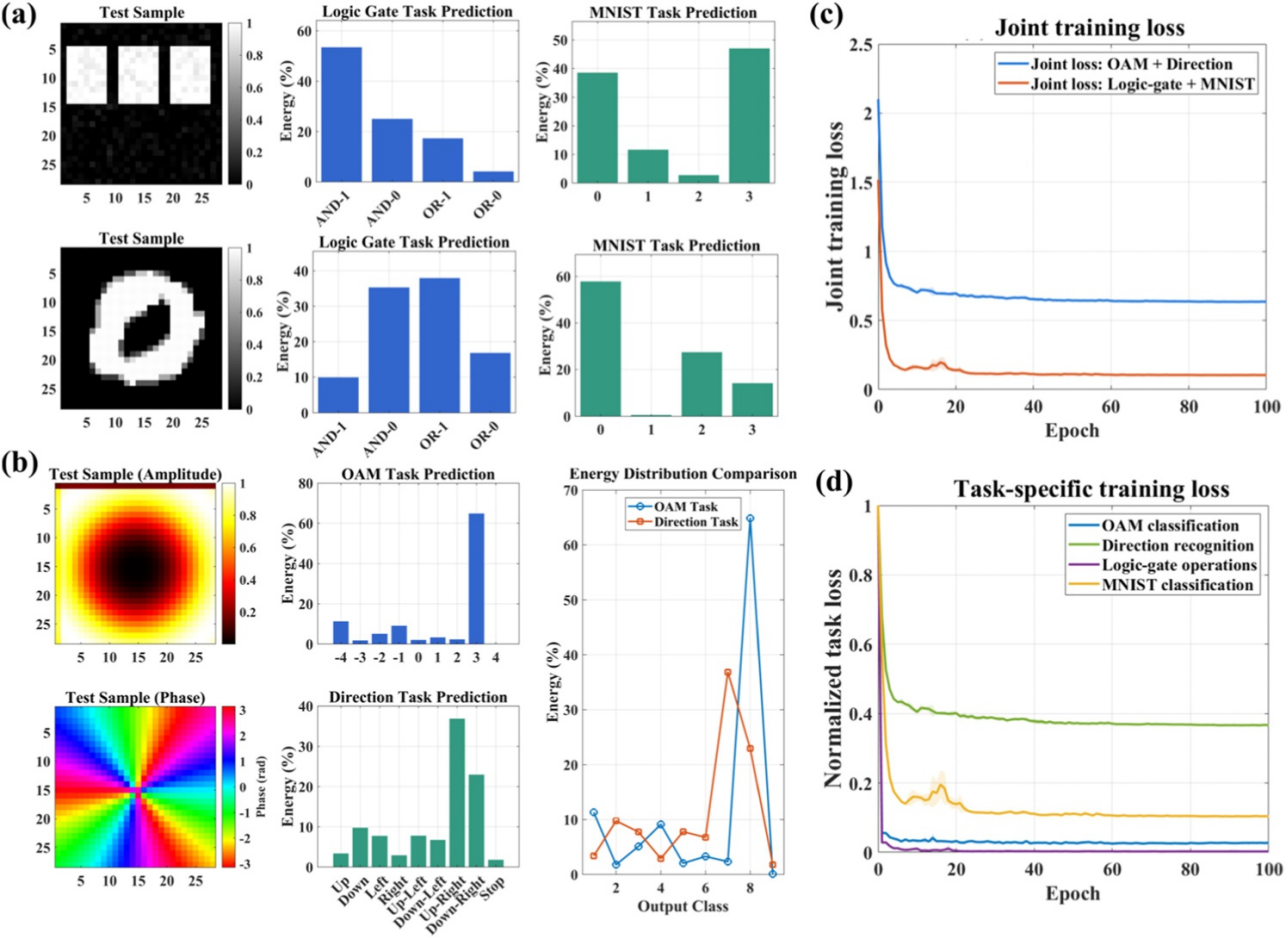
Task switching via interlayer spacing reconfiguration. (a) At 37.2 GHz, identical inputs yield distinct outputs for logic‐gate operations (*d*_1_) and MNIST classification (*d*_2_). (b) At 22 GHz, the same OAM input produces different responses for OAM classification (*d*_1_) and direction recognition (*d*_2_), demonstrating clear task discrimination. (c) Joint training losses of the OAM‐classification–direction‐recognition and logic‐gate–MNIST task groups. (d) Corresponding task‐specific training losses normalized by their respective initial values.

The convergence behavior and training stability of the two frequency‐specific dual‐task networks are further presented in Figure 7c,d. Each dual‐task network was independently trained using three random seeds, and the solid curves and shaded regions represent the mean loss and one standard deviation, respectively. As shown in Figure 7c, both joint training losses decrease rapidly during the initial stage and subsequently converge to stable plateaus. Figure 7d shows the task‐specific losses normalized by their respective initial values. All four losses decrease consistently during training without persistent oscillation or divergence. The direction‐recognition loss converges to a higher level than the other task losses, which is consistent with the greater similarity among several directional classes. The relatively narrow standard‐deviation bands indicate limited variation among the three independent runs and support the stability of the proposed multitask optimization under the tested initialization conditions.

It should be emphasized that the contribution of the proposed architecture lies in using several calibrated propagation distances as discrete computational states and jointly optimizing these states together with frequency multiplexing and shared phase modulation. Consequently, the same static dual‐frequency metasurface stack can implement multiple heterogeneous mappings without replacing or reprogramming its phase layers.

To provide a direct comparison with conventional diffractive deep neural networks, four single‐task D^2^NNs were independently trained for OAM classification, direction recognition, logic‐gate operations, and MNIST classification. Each baseline employed the same number of diffractive layers, spatial dimensions, operating frequency, nominal propagation distance, dataset division, and optimization settings as the corresponding task channel of the proposed DD^2^NN.

As shown in Table 2, the conventional single‐task networks achieved testing accuracies of 99.9%, 88.0%, 99.7%, and 96.8% for the four tasks, respectively, whereas the proposed DD^2^NN achieved 99.7%, 86.4%, 98.8%, and 95.4%. The corresponding accuracy differences were 0.2, 1.6, 0.9, and 1.4 percentage points. The average accuracy decreased only from 96.1% for the independently optimized baselines to 95.1% for the proposed shared architecture.

**TABLE 2:** Comparison between conventional single‐task D^2^NNs and the proposed DD^2^NN.

Task	Conventional D^2^NN (%)	Proposed DD^2^NN (%)	Accuracy difference (%)
OAM classification	99.9	99.7	−0.2
Direction recognition	88.0	86.4	−1.6
Logic‐gate operations	99.7	98.8	−0.9
MNIST classification	96.8	95.4	−1.4
Average	96.1	95.1	−1.0

More importantly, four conventional single‐task D^2^NNs would require four independent physical stacks. With three intermediate phase layers in each network, this corresponds to 12 separately fabricated diffractive layers. In contrast, the proposed DD^2^NN integrates the four tasks into one stack containing three shared dual‐frequency metasurface layers. The required number of physical phase layers is therefore reduced by 75%, whereas the number of supported tasks per physical stack is increased by a factor of four. These results show that the principal advantage of the proposed architecture lies in heterogeneous‐task integration and passive‐hardware reuse while maintaining performance close to that of independently optimized single‐task networks. Additional benchmarks involving complete ten‐class MNIST recognition and distance‐conditioned split‐MNIST classification are provided in Supporting Information S1: Note S4 and Table S1.

To bridge the gap between scalar diffraction modeling and physical reality, we performed full‐wave electromagnetic simulations using CST Microwave Studio. Unlike the scalar model, these simulations account for vector electromagnetic effects, near‐field coupling between adjacent unit cells, and material losses, providing a rigorous test of the design's physical viability.

As shown in Figure 8, the full‐wave results exhibit clear energy focusing at the designated output positions across all four tasks. For OAM classification at *f*_1_ = 22 GHz with *d*_1_ spacing (Figure 8a), distinct focal spots appear at the corresponding detector positions for each mode from *l* = −4 to +4. The direction recognition task under the *d*_2_ configuration (Figure 8b) similarly shows well‐defined output distributions for all 9 categories. At *f*_2_ = 37.2 GHz, both logic‐gate operations (Figure 8c) and MNIST digit recognition (Figure 8d) demonstrate successful classification, with concentrated energy at the correct output regions. These full‐wave results confirm that the designed dual‐frequency metasurface preserves the task‐dependent focusing behavior under realistic electromagnetic conditions, including vector‐field effects, intercell coupling, and material losses.

**FIGURE 8:**
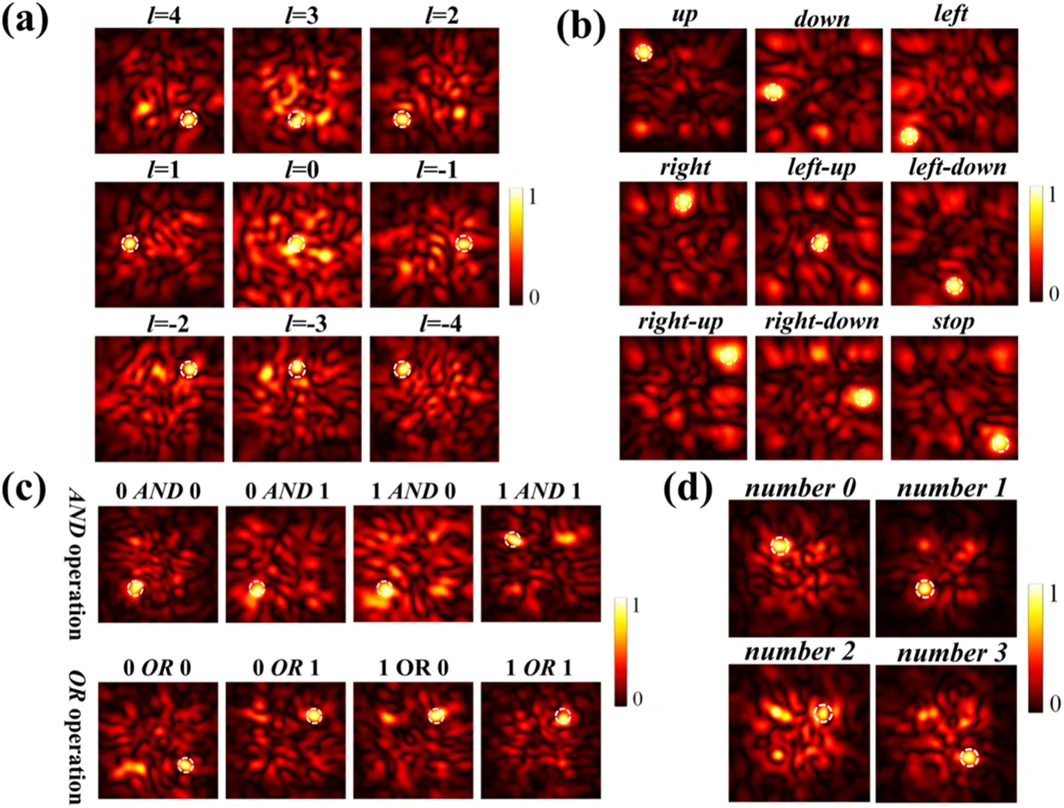
Full‐wave simulation results for all four tasks: (a) OAM classification (*l* = −4 to +4) at 22 GHz with *d*_1_, (b) direction recognition at 22 GHz with *d*_2_, (c) logic‐gate operations at 37.2 GHz with *d*_1_, and (d) MNIST digit recognition (0–3) at 37.2 GHz with *d*_2_.

To experimentally assess the feasibility of the proposed architecture, a physical prototype was fabricated using standard printed circuit board (PCB) technology, with the three‐layer metasurface manufactured according to the optimized phase distributions. As shown in Figure 9a–b, the experimental setup was constructed in a microwave anechoic chamber to minimize environmental noise. A vector network analyzer (VNA) was connected to transmitting and receiving horn antennas for field measurement. The input layer encodes task‐specific patterns, whereas a precise linear translation stage enables adjustment of the interlayer distances between the *d*_1_ (60 mm) and *d*_2_ (80 mm) configurations for task switching. Representative fabricated input samples are displayed in Figure 9c, including a directional arrow for movement recognition, a logic‐gate input pattern, an MNIST digit template, and a phase mask for OAM beam generation.

**FIGURE 9:**
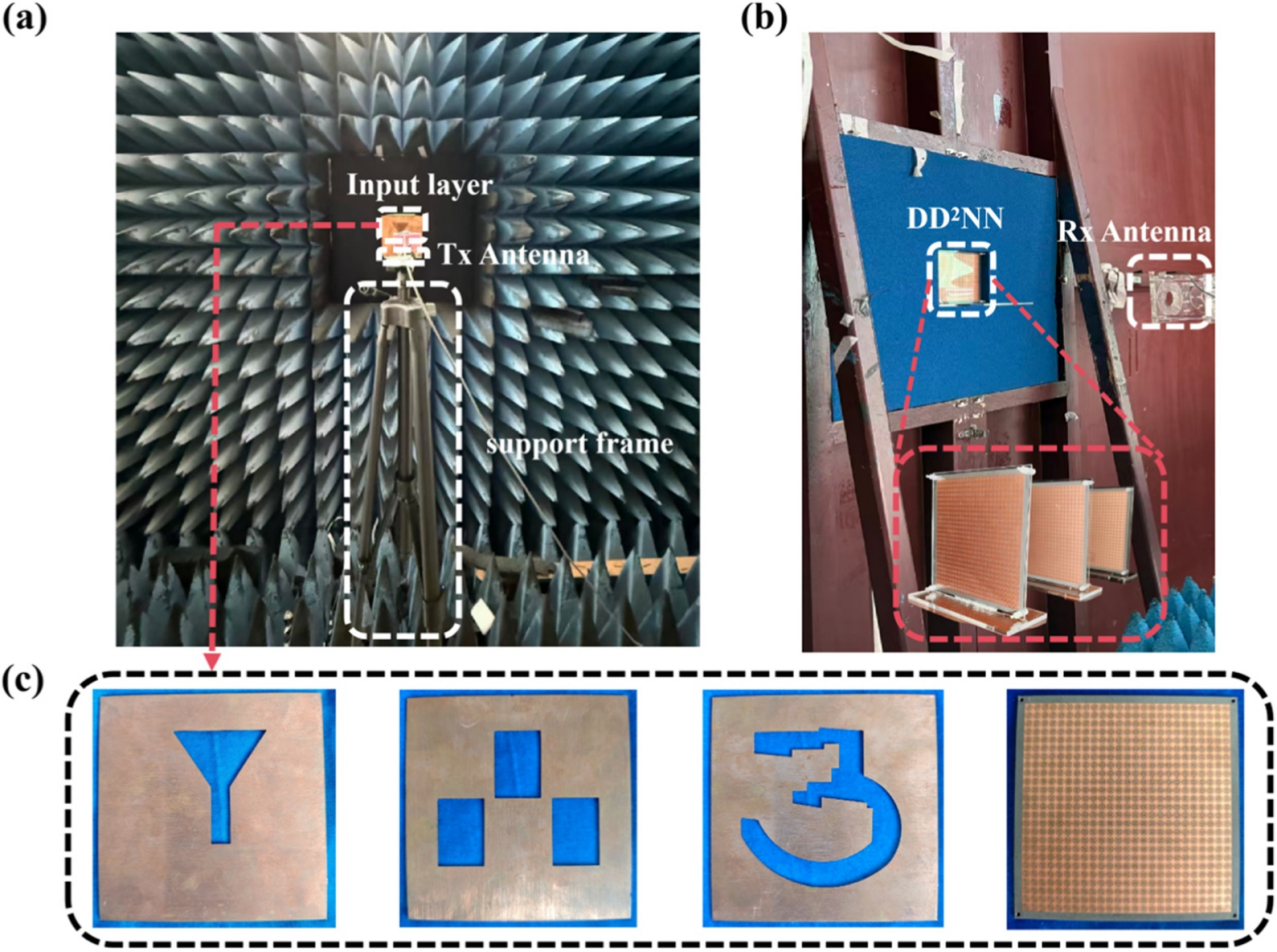
Experimental setup and fabricated samples. (a) Measurement environment in an anechoic chamber, showing the input layer, Tx antenna, and support frame. (b) DD^2^NN metasurface system with Rx antenna for output detection. (c) Fabricated input samples for the four tasks.

The experimental results for the 37.2‐GHz tasks are presented in Figure 10a–b. For logic‐gate operations, the measured energy distributions clearly distinguish between different logic states, with experimental values closely matching simulated predictions. The MNIST classification demonstrates successful digit recognition, where the energy for Digit “3” is predominantly concentrated at the correct output category. Additionally, Figure 10c shows the OAM classification result at 22 GHz, where the *l* = −2 mode is correctly identified, with the highest energy appearing at the corresponding output position.

**FIGURE 10:**
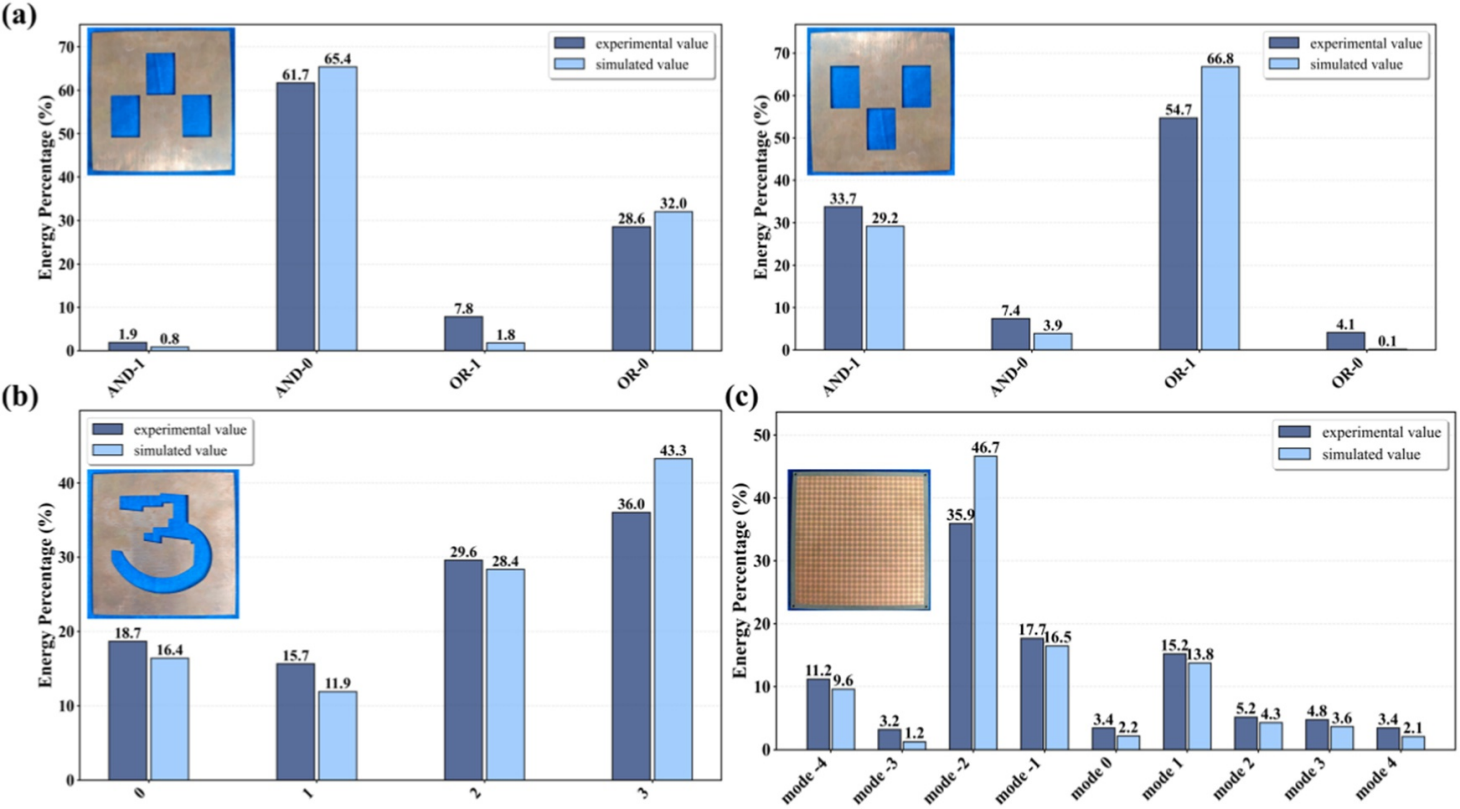
Experimental results at 37.2 GHz and OAM verification at 22 GHz. (a) Logic‐gate operations: measured versus simulated energy distributions for AND/OR inputs. (b) MNIST Digit “3” recognition, with a clear energy peak at the correct output. (c) OAM mode *l* = −2 identification at 22 GHz.

Figure 11 presents the direction recognition results at 22 GHz. Four representative directional inputs were tested, and the measured energy distributions show good agreement with full‐wave simulations. Despite the increased complexity of the 9‐class classification task, the system consistently routes the maximum energy to the correct directional category, validating the effectiveness of the distance‐encoding mechanism in practical implementations.

**FIGURE 11:**
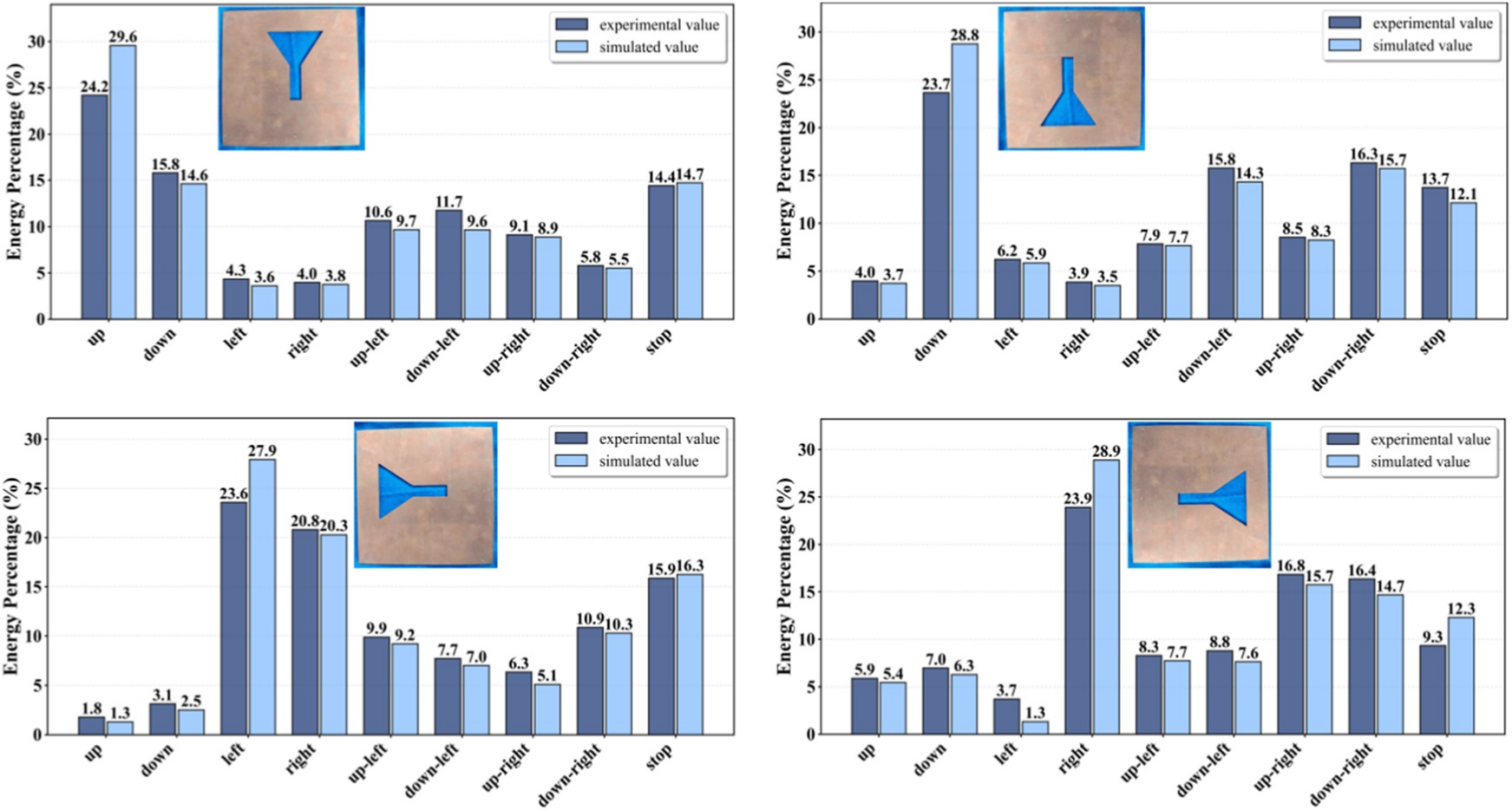
Direction recognition at 22 GHz: energy distributions for four representative inputs (up, down, left, and right arrows).

These measurements provide proof‐of‐concept evidence that the fabricated system can produce task‐dependent energy routing under selected frequency–distance configurations. Because only selected inputs were measured, these results should not be interpreted as experimental estimates of the dataset‐level classification accuracies reported from the scalar simulations.

Although the experimental results align well with both numerical and electromagnetic simulations, we observed a slight reduction in absolute focusing efficiency and contrast ratio. These discrepancies are primarily attributed to fabrication tolerances in the PCB etching process, minor alignment errors during the manual adjustment of layer spacing, and the inherent dielectric losses of the F4B substrate. Nevertheless, these representative measurements provide direct experimental evidence of the feasibility of task switching in the proposed joint distance–frequency encoding architecture.

To quantitatively evaluate the sensitivity of the proposed DD^2^NN to positioning errors, an additional tolerance analysis was performed after training, with all optimized phase distributions frozen. The longitudinal displacement was scanned from −12 to +12 mm with a step of 1 mm, whereas the lateral displacement was scanned from −4 to +4 mm with a step of 0.5 mm. For a longitudinal perturbation, each of the three internal diffractive layers was individually displaced by *Δz*, thereby increasing one adjacent propagation distance and decreasing the other by the same amount. For lateral perturbations, each layer was independently translated along the *x*‐ and *y*‐directions. At each displacement, the complete testing dataset was evaluated. The longitudinal curves represent the worst‐case accuracy among the three internal layers, and the lateral curves represent the worst case among the three layers and the two transverse directions.

At each displacement, one internal diffractive layer was perturbed at a time, and the worst‐case testing accuracy among the three layers was reported. The horizontal dashed lines denote the task‐specific thresholds corresponding to a five‐percentage‐point reduction from the nominal testing accuracy.

The tolerance was defined as the largest conservative symmetric interval within which the testing accuracy decreased by no more than 5 percentage points from its nominal value. As shown in Figure 12a–d, at 22 GHz, the OAM‐classification and direction‐recognition tasks both exhibited longitudinal tolerances of approximately ± 7.0 mm, whereas their lateral tolerances were approximately ± 2.5 and ± 1.5 mm, respectively. At 37.2 GHz, the longitudinal tolerances were approximately ± 7.0 mm for logic‐gate operations and ± 9.0 mm for MNIST classification, whereas both tasks exhibited lateral tolerances of approximately ± 2.5 mm.

**FIGURE 12:**
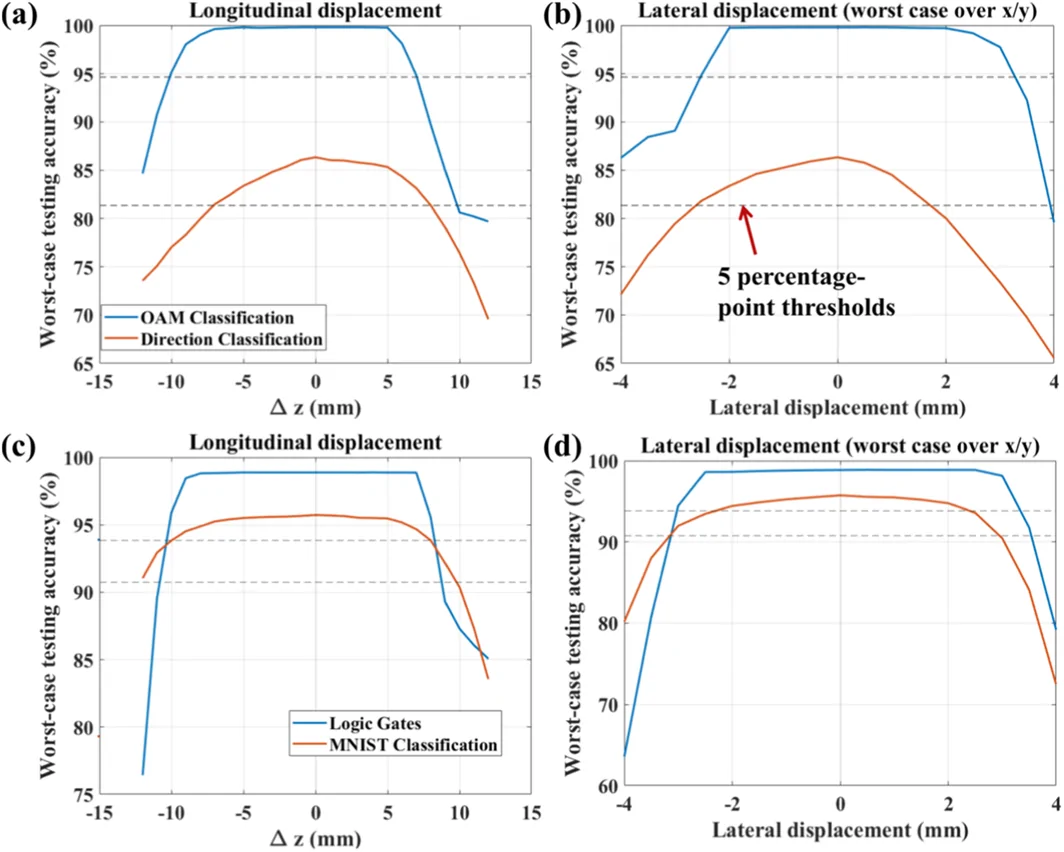
Numerical positioning‐tolerance analysis of the proposed DD^2^NN. (a, b) Longitudinal and lateral tolerances for OAM classification and direction recognition at 22 GHz. (c, d) Corresponding tolerances for logic‐gate operations and MNIST recognition at 37.2 GHz. Dashed lines indicate accuracy thresholds 5 percentage points below the nominal values.

Considering all four tasks, the conservative system‐level tolerance was approximately ± 7.0 mm longitudinally and ± 1.5 mm laterally. The lateral value corresponds to 0.375*P*, where *p* = 4.0 mm is the unit‐cell period. Although the output‐energy concentration and contrast gradually deteriorate with increasing displacement, the target detector remains dominant for the large majority of test samples, such that the overall testing accuracy stays within 5 percentage points of its nominal value. The broad accuracy plateaus observed for some tasks arise from the discrete maximum‐energy decision rule and do not indicate that the underlying output field distributions are invariant. These results demonstrate that the trained DD^2^NN exhibits finite numerical tolerance to practical positioning errors. This behavior is consistent with the intended effect of the alignment‐aware perturbation augmentation.

## 5 Conclusion

In this work, we proposed a multitask diffractive deep neural network architecture enabled by joint distance and frequency encoding and provided representative experimental evidence of its physical feasibility. By identifying the interlayer propagation distance as a reconfigurable physical dimension and designing a dual‐frequency phase‐decoupled metasurface, we successfully integrated four distinct computational tasks into a single passive hardware system. The reported classification accuracies were obtained from scalar diffraction simulations evaluated on the corresponding testing datasets. Full‐wave electromagnetic simulations further supported the feasibility of the designed metasurface implementation. Representative measurements showed that changing the interlayer spacing altered the task‐dependent output‐energy distribution at a fixed operating frequency. These representative proof‐of‐concept observations across different functional domains indicate that distance and frequency can serve as complementary task‐encoding dimensions under the tested configurations. By eliminating the need for complex external switching hardware, our approach preserves the passive, energy‐efficient nature of diffractive computing. These results establish the feasibility of joint distance–frequency encoding for multitask passive diffractive computing in the microwave regime and suggest potential applications in radio‐frequency sensing. Extension of this concept to integrated nanophotonic platforms remains a prospective direction that would require addressing challenges associated with wavelength‐dependent metasurface design, material dispersion and losses, nanoscale fabrication and alignment, and precise interlayer spacing control.

## Author Contributions

**Zhengxuan Jiang:** conceptualization, methodology, data curation, investigation, validation, writing – original draft. **Zongmao Fei:** validation, data curation, methodology. **Wen Jiang:** validation, investigation. **Guowen Ding:** conceptualization, writing – review and editing, resources, project administration, funding acquisition, supervision. **Ke Chen:** writing – review and editing, resources. **Yijun Feng:** resources, writing – review and editing.

## Funding

This work was supported by the National Natural Science Foundation of China (Grant 62301262). This work was supported by the China Postdoctoral Science Foundation (Grant 2022M720063).

## Conflicts of Interest

The authors declare no conflicts of interest.

## Supporting information


Supporting Information S1

## Data Availability

The data that support the findings of this study are available from the corresponding author upon reasonable request.
