# Supplementary material for: Multitask Diffractive Deep Neural Networks Enabled by Joint Distance–Frequency Encoding
Source: Nanophotonics. 2026 Oct 30;15(21):e70342. doi: 10.1002/nap2.70342 (PMC13720897; doi:10.1002/nap2.70342)
Supplement: Supplementary file 1 [file NAP2-15-e70342-s001.docx]

**Supporting Information**

**Multi-Task Diffractive Deep Neural Networks Enabled by Joint Distance–Frequency Encoding**

Zhengxuan Jianga, Zhongmao Feia, Wen Jianga,Guowen Dinga,b,*, Ke Chenb, Yijun Fengb

^a^*Research Center of Applied Electromagnetics, Nanjing University of Information Science and Technology, Nanjing 210044, China*

^b^*School of Electronic Science and Engineering, Nanjing University, Nanjing 210093, China*

^*^*gwding@nuist.edu.cn*

**The supporting Information includes: Note S1-S4,Fig. S1,Table S1**

**Note S1：**

To enable a more transparent assessment of task difficulty and generalization, the dataset-construction, sample-variability, and train–test separation procedures are described in detail. For OAM classification, nine topological-charge classes from *l*=−4 to *l*=+4 were considered, with 5,000 samples generated for each class. Random in-plane rotations, transverse displacements of up to 5% of the input-grid width, and amplitude perturbations with a standard deviation of 0.02 were introduced. The resulting 45,000 samples were randomized and divided into 36,000 training samples and 9,000 testing samples without sample overlap.

The direction-recognition dataset contained nine categories: up, down, left, right, up-left, down-left, up-right, down-right, and stop. Each category contained 5,000 samples. The arrow-shaft thickness, arrowhead size, in-plane rotation, transverse position, spatial scale, and additive noise were varied independently. The rotation angle was sampled within ±10°, the transverse displacement within ±3 pixels, the scale factor from 0.8 to 1.2, the line thickness from 1 to 4 pixels, and the Gaussian-noise standard deviation was set to 0.1. The resulting samples were divided into 36,000 training samples and 9,000 disjoint testing samples.

The logic-gate dataset included all eight balanced combinations of the left binary input, operator type, and right binary input. These combinations were mapped to four output categories: AND-0, AND-1, OR-0, and OR-1. The training and testing sets were independently generated using random seeds 20261001 and 20261002. Each input/operator combination contained 4,000 training samples and 1,000 testing samples, giving 32,000 training samples and 8,000 testing samples. Random translations of up to ±1 pixel, isotropic scaling from 0.95 to 1.05, and additive Gaussian noise with a standard deviation of 0.05 were applied.

**Note S2：**

The Pancharatnam–Berry phase introduced by an anisotropic meta-atom is directly related to its in-plane orientation angle. For the relevant circular-polarization-converted transmission component, the geometric-phase variation ideally satisfies

$\Delta\phi_{PB}=\pm2\Delta\theta$	(S1)

where *θ* denotes the in-plane rotation angle of the anisotropic resonator. The sign depends on the handedness of the incident and transmitted circularly polarized components, whereas the magnitude of the ideal phase slope is 2° per degree of physical rotation.

The proposed dual-frequency meta-atom contains an outer double split-ring resonator (DSRR) and an inner split-ring resonator (SRR), whose orientation angles are denoted by *α* and *β*, respectively. To examine the frequency-selective PB-phase modulation of these two resonators, the transmitted phases were extracted at the two operating frequencies while independently sweeping *α* or *β* from 0° to 150°. During the sweep of one orientation angle, the remaining geometric parameters and the orientation of the other resonator were kept unchanged.

Because the simulated phase is initially returned within a principal 360° interval, phase discontinuities may occur when the response crosses the 180° boundary. The phase data were therefore unwrapped before evaluating their linearity. The unwrapped phase was fitted using

$\phi_{fit}(\theta)=k\theta+b$	(S2)

where *k* is the fitted phase slope and *b* is a constant phase offset. The coefficient of determination was calculated as

$R^{2}=1-\frac{\sum_{i} {[\phi_{u,i}-\phi_{fit,i}]}^{2}}{\sum_{i} {[\phi_{u,i}-\bar{\phi_{u}}]}^{2}}$	(S3)

where $\phi_{u,i}$is the unwrapped simulated phase, $\phi_{fit,i}$is the fitted phase, and $\bar{\phi_{u}}$ is the mean unwrapped phase.

**Fig. S1**. Rotation-angle-dependent phase responses of the dual-frequency metasurface unit cell.

As shown in Fig. S1, pecifically, for the outer DSRR rotation angle *α*, the phase response at 22 GHz varies approximately linearly from −135.1° at *α*=0° to 166.0° at *α*=150°. A linear fit gives a slope of approximately 2.02° per degree of rotation with *R^2^*=0.9996, in close agreement with the expected PB-phase relation $\left| \text{Δ}\phi\right|\approx2\text{Δ}\alpha$. In comparison, the phase variation induced by *α* at 37.25 GHz remains comparatively limited.

For the inner SRR rotation angle *β*, after phase unwrapping, the phase response at 37.25 GHz varies nearly linearly from approximately −36.4° at *β*=0° to 245.9° at *β*=150°. The fitted slope is approximately 1.89° per degree of rotation with *R^2^*=0.9967. Meanwhile, the corresponding 22 GHz cross response remains weak.

To further characterize the frequency-selective modulation performance of the proposed dual-frequency meta-atom, the cross-frequency phase and transmission-amplitude variations were quantitatively evaluated under independent rotations of the outer DSRR and inner SRR. The transmission responses were obtained from full-wave electromagnetic simulations, with the remaining structural parameters held constant during each rotation sweep.

The cross-frequency phase fluctuation was characterized by the half peak-to-peak phase excursion, defined as half the difference between the maximum and minimum unwrapped transmission phases over the sampled rotation states. The peak-to-peak amplitude variation was defined as the difference between the maximum and minimum linear transmission magnitudes. For the phase analysis, six rotation angles from 0° to 150° with an interval of 30° were considered.

When the outer DSRR rotation angle *α* was varied, the primary transmission phase at 22 GHz changed from −135.11° to 166.01°, corresponding to a phase tuning range of 301.12°. In comparison, the cross-frequency phase response at 37.25 GHz exhibited a peak-to-peak fluctuation of $\pm$13.64°. The corresponding transmission amplitude at 37.25 GHz varied from 0.85211 to 0.91548, giving a peak-to-peak linear amplitude variation of 0.06336, equivalent to approximately 0.62 dB.

Similarly, when the inner SRR rotation angle *β* was varied, the unwrapped primary transmission phase at 37.25 GHz changed from −36.37° to 245.94°, corresponding to a phase tuning range of 282.31°. Meanwhile, the cross-frequency phase variation at 22 GHz was limited to $\pm$2.80°. The corresponding transmission amplitude at 22 GHz ranged from 0.77415 to 0.81925, yielding a peak-to-peak linear amplitude variation of 0.04509, equivalent to approximately 0.49 dB.

The cross-frequency phase fluctuations are substantially smaller than the corresponding primary phase tuning ranges, indicating that the outer and inner resonators primarily control the low- and high-frequency phase responses, respectively. The relatively small cross-frequency transmission-amplitude variations provide additional evidence of frequency-selective modulation.

Nevertheless, the finite phase and amplitude variations indicate residual electromagnetic coupling between the two resonators. The proposed dual-frequency meta-atom therefore exhibits approximate frequency-selective phase decoupling rather than perfectly independent amplitude and phase responses. These characteristics support the realization of distinct phase-modulation profiles at the two operating frequencies, thereby facilitating the implementation of the proposed joint distance–frequency encoding strategy.

**Note S3：**

The phase modulation mechanism of the proposed dual-frequency metasurface is based on the Pancharatnam–Berry (PB) geometric phase, which originates from the rotation of anisotropic electromagnetic structures. For a transmissive anisotropic meta-atom, the polarization response can be characterized by its Jones transmission matrix in the Cartesian linear-polarization basis:

$$\mathbf{T}_{0}=\left( \begin{matrix} t_{xx} & t_{xy} \\ t_{yx} & t_{yy} \end{matrix} \right)$$

where $t_{xx}$ and $t_{yy}$ denote the co-polarized transmission coefficients, while $t_{xy}$ and $t_{yx}$ represent the cross-polarized transmission coefficients of the reference structure.

When the anisotropic structure is rotated by an in-plane angle $\theta$, its transmission matrix transforms according to the corresponding coordinate rotation. By expressing the rotated transmission response in the circular-polarization basis, the polarization-converted transmission coefficients can be written as

$t_{\mathrm{LR}}\left( \theta\right)=\frac{1}{2}\left[ \left( t_{\mathrm{xx}}-t_{\mathrm{yy}} \right)-j\left( t_{\mathrm{xy}}+t_{\mathrm{yx}} \right) \right]e^{-j2\theta}$	(S4)

$t_{\mathrm{RL}}\left( \theta\right)=\frac{1}{2}\left[ \left( t_{\mathrm{xx}}-t_{\mathrm{yy}} \right)+j\left( t_{\mathrm{xy}}+t_{\mathrm{yx}} \right) \right]e^{+j2\theta}$	(S5)

where $t_{LR}$ and $t_{RL}$ denote the transmission coefficients between opposite circular-polarization states under the adopted polarization convention. The coefficients $t_{xx}$, $t_{yy}$, $t_{xy}$, and $t_{yx}$ correspond to the unrotated reference structure.

Equations (S4) and (S5) indicate that the circular-polarization-converted transmission components acquire additional phase factors of $e^{+j2\theta}$ and $e^{-j2\theta}$. Consequently, the rotation-dependent PB geometric-phase shifts satisfy

$\Delta\phi_{\mathrm{LR}}=-2\theta, \Delta\phi_{\mathrm{RL}}=+2\theta$	(S6)

The sign of the geometric phase depends on the incident and transmitted circular-polarization handedness, whereas the magnitude of the phase variation is twice the physical rotation angle. Thus, an ideal rotation of 180° provides a complete 360° geometric-phase modulation range.

For an ideal anisotropic half-wave element, efficient circular-polarization conversion can be achieved when the two principal-axis transmission coefficients have approximately equal magnitudes and a phase difference of 180°. Under the ideal lossless condition, the co-polarized circular component is suppressed, and the transmitted energy is converted into the opposite circular-polarization component. The resulting geometric phase can therefore be controlled through structural rotation without requiring a corresponding change in the transmission amplitude.

In the proposed dual-frequency meta-atom, the outer double split-ring resonator (DSRR) and inner split-ring resonator (SRR) provide two distinct rotational degrees of freedom. The outer DSRR rotation angle *α* primarily controls the transmission phase at 22 GHz, whereas the inner SRR rotation angle *β* primarily controls the phase at 37.2 GHz. Under the selected polarization and phase-reference conventions, the corresponding ideal phase modulation relationships are approximately $\phi_{1}=2\alpha$ and $\phi_{2}=2\beta$, respectively.

Therefore, the desired phase distributions $\phi_{1}(x,y)$ and $\phi_{2}(x,y)$, obtained from the frequency-specific diffractive-network optimization, can be mapped to the physical rotation angles according to

$$\alpha\left( x,y \right)=\frac{\phi_{1}\left( x,y \right)}{2}, \beta\left( x,y \right)=\frac{\phi_{2}\left( x,y \right)}{2}$$

which provides the physical basis for the factor (1/2) in Equation (5) of the main manuscript. These relationships are understood with the appropriate phase reference and angular periodicity.

It should be emphasized that the ideal Jones-matrix rotation relationship describes a rigidly rotated anisotropic element. In the proposed nested dual-resonator structure, independently rotating one resonator while keeping the other fixed may introduce residual electromagnetic coupling, leading to deviations from the ideal PB-phase response. Accordingly, the factor-of-two relationship serves as the approximate geometric-phase design principle, while the actual frequency-selective phase behavior is evaluated using full-wave electromagnetic simulations.

The rotation-angle-dependent phase responses and corresponding linear-fitting results are presented in Supplementary Note S2 and Fig. S1. These numerical results further support the applicability of the PB geometric-phase mechanism to the proposed dual-frequency metasurface.

**Note S4：**

Table S1. Complete ten-class MNIST and logic-gate benchmark.

To further evaluate the computational capability using a standard full-scale benchmark, the complete ten-class MNIST dataset was considered. The independently optimized single-task MNIST D²NN achieved 85.29% testing accuracy, whereas the proposed shared DD²NN achieved 84.78% while simultaneously maintaining 98.82% accuracy on the logic-gate task. The performance reduction relative to the single-task MNIST reference was therefore only 0.51 percentage points. A frequency-only shared D²NN achieved 83.77% MNIST accuracy and 98.11% logic-gate accuracy. Introducing the distance-encoding degree of freedom therefore improved the MNIST accuracy by 1.01 percentage points relative to the frequency-only shared baseline under matched conditions.

An auxiliary distance-conditioned split-MNIST experiment was also performed. Digits 0–4 and digits 5–9 were assigned to two calibrated distance states, yielding accuracies of 95.79% and 89.50%, respectively, with a sample-number-weighted value of 92.74%. The combined accuracy was calculated by weighting the two subset accuracies according to their respective test sample counts. Because selection of the appropriate distance state requires prior knowledge of the digit subset, this result represents an oracle-conditioned ablation experiment and is not directly compared with the standard end-to-end ten-class MNIST result. Nevertheless, it demonstrates that different propagation-distance states can support distinct classification mappings within the same data domain.
